# Between a bug and a hard place: *Trypanosoma cruzi* genetic diversity and the clinical outcomes of Chagas disease

**DOI:** 10.1586/14787210.2015.1056158

**Published:** 2015-07-10

**Authors:** Louisa A Messenger, Michael A Miles, Caryn Bern

**Affiliations:** ^a^ ^1^Department of Pathogen Molecular Biology, Faculty of Infectious Tropical Diseases, London School of Hygiene and Tropical Medicine, London, UK; ^b^ ^2^Global Health Sciences, Department of Epidemiology and Biostatistics, School of Medicine, University of California San Francisco, San Francisco, CA, USA

**Keywords:** cardiomyopathy, • Chagas disease, congenital transmission, diagnostics, genetic diversity, oral outbreaks, reactivation, treatment

## Abstract

Over the last 30 years, concomitant with successful transnational disease control programs across Latin America, Chagas disease has expanded from a neglected, endemic parasitic infection of the rural poor to an urbanized chronic disease, and now a potentially emergent global health problem. *Trypanosoma cruzi* infection has a highly variable clinical course, ranging from complete absence of symptoms to severe and often fatal cardiovascular and/or gastrointestinal manifestations. To date, few correlates of clinical disease progression have been identified. Elucidating a putative role for *T. cruzi* strain diversity in Chagas disease pathogenesis is complicated by the scarcity of parasites in clinical specimens and the limitations of our contemporary genotyping techniques. This article systematically reviews the historical literature, given our current understanding of parasite genetic diversity, to evaluate the evidence for any association between *T. cruzi* genotype and chronic clinical outcome, risk of congenital transmission or reactivation and orally transmitted outbreaks.

## Background

Chagas disease is the most important parasitic infection in Latin America, affecting an estimated 5–6 million individuals, with a further 70 million at risk [1]. The geographical range of the etiological agent, *Trypanosoma cruzi* (Kinetoplastida: Trypanosomatidae), extends from the southern USA to Argentinean Patagonia, where it is transmitted by more than 100 species of hematophagous triatomine bugs (Hemiptera: Reduviidae: Triatominae) [2,3] to at least eight orders of domestic, synanthropic and sylvatic mammalian hosts [4]. Human disease occurs when infected triatomine feces enter through intact mucosa or abraded skin [5]. Oral transmission is an important secondary infection route, responsible for regional microepidemics of acute Chagas disease in areas often devoid of domestic triatomine species, for example, the Amazon Basin [6]. In recent years, a significant proportion of the infected population has emigrated from rural areas, leading to the urbanization of Chagas disease in endemic countries as well as internationally [7]. Chagas disease is now considered an emergent global public health problem associated with congenital transmission [8], blood transfusions [9] and organ transplantations [10].

Following *T. cruzi* exposure, human infection begins with an acute phase, lasting up to 3 months, during which circulating trypomastigotes can be visualized in peripheral blood films or buffy coat smears. Most individuals are asymptomatic or present with a non-specific, self-limiting febrile illness [8]. Mortality during the acute phase is rare (<1% of cases) and may result from severe myocarditis, pericardial effusion and/or meningoencephalitis [11]. Acute mortality occurs more frequently in infants and immunocompromised patients than other infected persons. Even in the absence of treatment, the acute phase spontaneously resolves in most individuals [8,11].

Chronic infection is initially asymptomatic and the majority of patients will remain clinically indeterminate for life. However, over a period of 10–30 years, approximately 20–30% of infected individuals will develop irreversible, potentially fatal cardiac syndromes (chronic chagasic cardiomyopathy [CCM]) and/or dilatation of the GI tract (megacolon or megaesophagus) [11]. Early CCM is typically characterized by conduction system abnormalities, particularly right bundle branch block and/or left anterior fascicular block, and premature ventricular contractions [12]. More advanced manifestations include ventricular tachycardia, high-degree atrioventricular block and progressive dilated cardiomyopathy with congestive heart failure [13]. Sudden death accounts for 30–65% of CCM-related mortality and can affect patients with end-stage heart disease as well as those who were previously asymptomatic [14]. Gastrointestinal (GI) megasyndromes are rarer than cardiac sequelae, resulting from denervation, decreased motility, sphincter dysfunction and eventual luminal dilatation of the esophagus and/or colon [15]. However, the prevalence of different clinical forms, especially digestive disease, varies considerably between geographical regions [16,17].

## Clinical staging of CCM

Multiple expert committees have published guidelines for the standard evaluation of patients with chronic *T. cruzi* infection [18–20]. All recommend thorough history and physical examination, and at a minimum, a 12-lead ECG. Some committees also recommend echocardiograms and/or barium studies of the esophagus and colon. If all of these are normal, the patient is considered to have the indeterminate form of Chagas disease (IND). The expert committee convened by the US CDC advised that barium studies only be performed if the patient reported GI symptoms, based on the low prevalence of the digestive form of Chagas disease in Mexico and Central America, the source of most infected individuals in the USA [18]. This committee also recommended against performing echocardiograms on patients with no cardiac symptoms or ECG abnormalities, because significant abnormal findings on echocardiogram are rare in the absence of other indications of Chagas cardiomyopathy.

Several systems have been used to characterize the severity of CCM for clinical staging and epidemiological studies. The most commonly cited schemes are the modified Kuschnir classification, the Los Andes classification and the more recent system that incorporates the American College of Cardiology/American Heart Association criteria for congestive heart failure staging [21–24]. All use similar general criteria, including specific ECG abnormalities plus chest radiography or echocardiogram to provide measures of left ventricular size and/or ejection fraction (see comparative table in [18]). However, many investigators add other modifications based on their own clinical experience, to provide finer scale classification or increase the specificity of staging [25,26]. Many ECG findings that are listed in these schemes to define early CCM, such as low voltage and right bundle branch block, occur in other cardiac diseases and are fairly common in older age groups, independent of *T. cruzi* infection status [27]. Others, such as moderate bradycardia and incomplete right bundle branch block, are normal variants in healthy young people [28]. While the most severe stage in any of the common classification schemes (equivalent to advanced congestive heart failure) is highly predictive of mortality [29], there is no obvious method to verify the clinical or epidemiological validity of criteria for the earlier stages. The use of heterogeneous classification schemes directly impedes comparisons of epidemiological studies of CCM severity and prevalence, and meta-analyses of the relationship between Chagas disease pathogenesis and *T. cruzi* strain diversity.

## A brief history of *T. cruzi* taxonomy

*T. cruzi* is an ancient parasite, estimated to have diverged from its most recent common ancestor 3–4 million years ago [30], and as such, displays considerable genetic diversity. Current international consensus recognizes a minimum of six stable genetic lineages: TcI–TcVI [31]. A potential seventh bat-restricted genotype (TcBat), with genetic affiliations to TcI, has recently been reported in Central and northern South America [32–34]. Historically, the taxonomy of *T. cruzi* has been hindered by a lack of standardized molecular typing methods and the use of various alternative nomenclatures (Table 1) [35].

**Table 1.** **An overview of *Trypanosoma cruzi* historical and contemporary nomenclatures.**

**Term**	**Technique**								**Ref.**
DTU (current nomenclature)		**I**	**II**	**III**	**IV**	**V**	**VI**		[31]
	SL-IR, 24Sα rRNA, 18S rRNA, Cytb, Histone H2B, ITS1 rDNA, 18S rRNA, gGAPDH							TcBat	[32–34]
Putative intra-TcI subdivisions	SL-IR	Ia, Ib, Ic, Id, Ie							[48–51]
	MLMT, mtMLST	I_DOM_							[52,53,70]
DTU^†^	MLEE, RAPD, 24Sα rRNA, 18S rRNA	I	IIb	IIc	IIa	IId	IIe		[45,46]
Zymodeme	MLEE	ZI	ZII	ZIII/ZI ASAT	ZIII	Bolivian ZII	Paraguayan ZII		[16,36,37,128,290–292]
	MLEE	ZB	ZA	ZC	ZD	ZB	ZB		[293,294]
	MLEE	Z2, 5, 7, 10 or 12	Z4 or 11			Z1, 3 or 9			[295]
	ITS-RFLP, 24Sα rRNA			ZIII-A	ZIII-B				[296]
Biodemes	MLEE	III	II			I	I		[171]
Clonet	MLEE	1–25	30–34	35–37	27–29	38, 39	40–43		[38]
*Trypanosoma cruzi*		*T. cruzi* I	*T. cruzi* II	*T. cruzi*	*T. cruzi*	*T. cruzi*	*T. cruzi*		[44]
Lineage	24Sα rRNA, SL-IR, RAPD	2	1			1/2	1/2		[41]
	24Sα rRNA, SL-IR	2	1	2′	2′	1	1		[42,43]
Clade	TR, DHFR-TS, COII-ND1	A	C	B	D	B + C	B + C		[60]
Riboclades	24Sα rRNA, 18S rRNA	2	1	3	4	3	1		[297]
Haplogroups	MLMT, 24Sα rRNA, COII, ND1, Cytb	ZZ	YY	XX		XY	XY		[54]
	TcMSH2	A	C				B		[298]
Group	Karyotyping	C	B		D	A	A		[154]
	SL-IR	II	I		I	I	I		[299]
Reference strain		Sylvio X10/1	Esm cl3	M5631 cl5	CanIII cl1	Sc43 cl1	CL Brener		

^†^Proposed by [47]. DHFR-TS: Dihydrofolate reductase-thymidylate synthase; gGAPDH: Glyceraldehyde 3-phosphate dehydrogenase; MLEE: Multilocus enzyme electrophoresis; MLMT: Multilocus microsatellite typing; mtMLST: Maxicircle multilocus sequence typing; RAPD: Random amplification of polymorphic DNA; RFLP: Restriction fragment length polymorphism; SL-IR: Spliced-leader intergenic region; TR: Trypanothione reductase.

The earliest attempts to characterize *T. cruzi* strain variation, based on multilocus enzyme electrophoresis (MLEE), classified isolates into three major groups or ‘zymodemes I, II and III’ [36,37], which were later subdivided into 43 ‘clonets’ [38]. Subsequent genotyping of additional strains using MLEE [39], random amplification of polymorphic DNA [40] and nuclear loci [41–43] grouped isolates into two major lineages, designated *T. cruzi* I and *T. cruzi* II [44]. More recently, supported by MLEE and multilocus sequence typing, TcII was separated into TcIIa–e [45,46], which were latterly renamed as TcII–TcVI to remove any presumptive sublineage designations [31]. Each of the six former *T. cruzi* genetic lineages is now considered a discrete typing unit (DTU), defined as ‘a collection of strains that are genetically more closely related to each other than to any other strain and that share one or several specific characters’ [47]. However, the criteria for division, number of subgroups, and their precise biological and evolutionary relevance are still a popular subject of debate [48–57].

The principal reproductive mode of *T. cruzi*, in particular the relative contributions of clonality and sexuality to parasite population structures and the evolution of discrete DTUs, is also contentious [58,59]. DTUs TcI–TcIV form monophyletic clades and TcV and TcVI are known to be recent inter-lineage hybrids [30]. As such, TcI–TcIV are characterized by substantial allelic homozygosity, likely resulting from recurrent, dispersed, genome-wide gene conversion, while TcV and TcVI display natural heterozygosity and minimal distinction, sharing intact alleles from their parental progenitors (TcII and TcIII) [54,57,60–62].

## Molecular epidemiology of *T. cruzi*

Molecular epidemiology studies have made substantial progress defining the phylogeographical and ecological niche of each *T. cruzi* lineage (Table 2) [63]. Sylvatic DTU distribution data are still largely aggregated due to differences in ease of capture between reservoir species, paucity of supporting ecological information and our inability to genotype subpatent zoonotic infections [64].

**Table 2.** **An overview of ecotopes, sylvatic vectors/hosts, geographical distributions and clinical associations of the major *Trypanosoma cruzi* discrete typing units.**

**DTU**	**Ecological niche**	**Domestic vectors**	**Sylvatic vectors**	**Sylvatic hosts**	**Geographical distribution**	**Clinical forms of human Chagas disease**	**Ref.**
TcI	Primary: lowland tropical arboreal Secondary: arid terrestrial	*Triatoma dimidiata* (Central America), *Rhodnius, Panstrongylus* (Brazil, Venezuela, Colombia) *Triatoma* (Peru, Bolivia)	Primary: *Rhodnius* spp. Secondary: *Triatoma, Panstrongylus*	Primary: Arboreal marsupials (*Didelphis*), primates, caviomorphs Secondary: Terrestrial rodents (*Phyllotis ocilae, Akodon boliviensis*)	Primary: southern USA, Central and South America Secondary: central Brazil and eastern Andean foothills	Cardiomyopathy Sporadic in Southern Cone	[48–51,65,66,70,72–74,76,78,88,185,187,300,301]
TcII	Rare in sylvatic cycles	*Triatomainfestans* *Panstrongylus megistus*	–	Atlantic forest primates	Atlantic/central Brazil and Southern Cone	Cardiomyopathy GI megasyndromes Congenital infections	[65,72,79–81,190,243,302],
TcIII	Terrestrial, fossorial, lowland, arid and tropical	–	*Panstrongylus geniculatus* *Panstrongylus lignarius* *Triatomarubrovaria*	Armadillos (*Dasypus novemcinctus, Euphractus sexcinctus, Chaetophractus*) marsupials (*Didelphis, Monodelphis*), rodents, carnivores	Northeastern Venezuela to Argentina	Rare in humans	[88–92,303]
TcIV	Arboreal with terrestrial transmission in North America	–	*Rhodnius, Panstrongylus, Triatoma*	Primates, *D. novemcinctus, Nasua nasua, Procyon lotor*	Southern USA and northern South America	Secondary agent in Venezuela Sporadic oral outbreaks in Brazilian Amazon	[16,66,68,93,95–99]
TcV	Rare in sylvatic cycles Putative peridomestic transmission among dogs	*T. infestans*	–	–	Principally Southern Cone, Gran Chaco Sporadic reports in Colombia and Ecuador	Cardiomyopathy GI megasyndromes Congenital infections	[73,82–84,87,135,168,200,304,305]
TcVI	Rare in sylvatic cycles Putative peridomestic transmission among dogs	*T. infestans*	–	–	Principally Southern Cone, Gran Chaco Sporadic reports in Colombia and Ecuador	Cardiomyopathy GI megasyndromes Congenital infections	[82–84,87]
TcBat	Not described	–	–	Chiroptera spp.	Panama, central and south-east Brazil and Colombia	One isolated human infection	[32–34,101]

DTU: Discrete typing unit; GI: Gastrointestinal.

In general, TcI, TcII, TcV and TcVI are most frequently isolated from domestic cycles and responsible for the majority of human infections. TcI has the widest distribution; it is the principal cause of Chagas disease in Colombia and Venezuela [65–67] and ubiquitous in the sylvatic environment [68,69], primarily circulating in arboreal ecotopes between *Didelphis* species and the triatomine tribe *Rhodniini* [70,71], with secondary terrestrial transmission among rodents and sylvatic *Triatoma* species in the inter-Andean valleys of Argentina, Bolivia, Peru and Chile [72–77]. Multiple molecular markers consistently identify high levels of genetic diversity within sylvatic TcI populations [48–51,70,78], and divergent, but genetically homogeneous, strains associated with domestic vectors and human infections [52,53,70].

By comparison, TcII, TcV and TcVI are less genetically diverse overall [30] and appear largely confined to domestic transmission cycles in southern parts of South America [63]. The sylvatic reservoirs of these three DTUs are not fully defined, although TcII has been increasingly isolated from primates in Brazil [64,79–81]; peridomestic dogs are emerging as potential reservoirs of TcV and TcVI in the Gran Chaco region [82–85]. The geographical range of TcV and VI appears to be more extensive than previously suggested, with isolated reports of these hybrid DTUs as far north as Ecuador [86] and Colombia [87]. TcIII has a dispersed terrestrial distribution that extends from northeastern Venezuela to Argentina, where it is transmitted by *Panstrongylus geniculatus* to *Dasypus novemcinctus* and other fossorial mammals [88–92]. TcIV is poorly understood, principally because several genotyping methods fail to distinguish this lineage from others, especially from TcIII [42,93,94]. However, TcIV is known to circulate sympatrically with TcI in wild primates, *Monodelphis* and *Dasypus* spp. in the Amazon [95] and raccoons and dogs in North America [96]. TcIV is also increasingly detected in human disease, as a secondary agent of Chagas disease in Venezuela [16,66], and in recent oral outbreaks in the Brazilian Amazon [95,93,97–99]. As of now, TcIII and TcIV have only been sporadically detected in domestic transmission cycles, but this may be attributable to undersampling and the limited sensitivity of some genotyping methods [100]. Finally, TcBat, a new, genetically divergent and potentially human-infective lineage [101], has been isolated from *Chiroptera* species across Panama [33], Brazil [32] and Colombia [34].

## *T. cruzi* clinical genotyping: perils & pitfalls

Establishing an association between *T. cruzi* genotype and clinical outcome is complicated by inherent biological features relating to parasite infection dynamics, as well as the limitations of our current repertoire of genotyping techniques. In humans [53,102–104], triatomine bugs [85,105,106] and mammalian reservoir hosts [88,107,108], mixed infections of distinct parasite clones are not exceptional but, in many cases, inevitable. In highly endemic areas, long-term inhabitants are repeatedly infected by multiple contacts with different triatomines [109], which in turn may have fed on various infected humans and/or mammals, depending on the local disease ecology.

Levels of intra-patient parasite multiclonality might be expected to increase proportionally with vector exposure. However, this assumes a constant force of infection, incomplete cross-genotypic immunity, and lack of genotype interaction (e.g., genotype displacement, reciprocal inhibition, potentiation or recombination) [110–114], transmission population bottlenecks (as observed in related trypanosomes [115]) or any additional mechanisms that might alter the establishment of secondary infections. The complexity of natural multiclonal parasite populations is largely unknown and our ability to detect them is restricted by genetic marker resolution [107,116]. The study of this phenomenon conventionally necessitates deriving biological clones from live parasite populations (by micromanipulation [117], limiting dilution [118], plating on semi-solid media [106] or FACS [116]), prior to genetic typing, which introduces a range of potential adaptation biases, discussed below.

Genotyping of *T. cruzi* can be performed either directly from clinical samples (blood or tissue biopsies) or following parasite isolation by hemoculture or xenodiagnosis. Due to the scarcity of parasites in peripheral blood, especially in chronically infected patients, the former method has limited sensitivity. The primary drawback associated with parasite isolation is selection bias for particular subpopulations, initially by preferential outgrowth due to faster dividing rates and/or culture media [55,119,120] and subsequently by loss of clonal diversity from serial maintenance in axenic culture or animals [121–126]. Hemoculturing is laborious; recovery rates are usually less than 30% among chronic patients [127] and almost entirely determined by parasite load and distribution within the starting sample. Xenodiagnosis, which can facilitate greater parasite recovery, has also been shown to vary depending on vector permissibility to local strains [128–130]. Furthermore, due to differential strain tropisms, circulating clones isolated by hemoculture or xenodiagnosis are often genetically distinct from those sequestered in tissues [102–104] and can vary even between sequential blood samples [131]. Together, these observations strongly suggest that intra-host parasite diversity is routinely underestimated.

A plethora of molecular genotyping techniques have been developed to characterize *T. cruzi* genetic diversity, with varying degrees of resolution, experimental ease, reproducibility, subjectivity and transferability (Table 3). Typing of genetic polymorphisms in conserved housekeeping genes can define major genetic lineages [41–43,45,46,61,132], while analysis of hypervariable loci such as microsatellites [70,89,133,134], or kinetoplast DNA (kDNA) minicircles [135–139] potentially allows identification of profiles specific to individual strains. Choice of typing methodology is principally determined by sample source, research objective and laboratory resources.

**Table 3.** **Overview of current and historical *Trypanosoma cruzi* genotyping methods.**

**Genotyping method**	**Method description**	**Example of genetic markers**	**Reproducibility b/w assays **	**Level of resolution**	**Reagent cost**	**Advantages**	**Disadvantages**	**Ref.**
MLEE	Measures differences in electrophoretic mobilities of isoenzymes	ASAT, ALAT, PGM, ACON, MPI, ADH, MDH, ME, ICD, 6PGD, G6PD, GD, PEP, GPI	High	DTU level Intra-lineage	Moderate	Easy visual interpretation Data amenable to numerical taxonomic analysis, for example, rates of similarity or genetic distance	Requires large quantities of parasite lysate from live strains	[16,128,173,293,294,306]
RAPD	Short random sequence primers used to amplify unknown DNA fragments to create unique band patterns	N/A	Low	DTU level	Low	Can be performed directly on field samples No prior sequence knowledge needed Unlimited number of primers Data amenable to numerical taxonomic analysis	Reproducibility issues Dominant markers may conceal heterozygosity Strain profiles may vary with DNA template amount and quality	[40,41,45,46]
kDNA-RFLP	RFLP analysis of kinetoplast mHVR	mHVR	Low	Intra-lineage	Low	Hypervariable markers Can produce strain-specific profiles	Requires isolation of kDNA from live parasites Strain profile inheritance may not be stable or correlate with nuclear typing Potential problems of contamination due to very high copy number	[136]
kDNA hybridization	Analysis of mHVR by radioactive probe hybridization	mHVR	Low	DTU-level Intra-lineage		Hypervariable markers Can produce strain-specific profiles	DNA probes may cross-react b/w DTUs Potential problems of contamination due to very high copy number	[307,308]
Karyotyping (aCSDI)	Comparison of chromosome size variation by PFGE separation and radioactive probe hybridization	1F8, cruzipan, FFAg6, Tc2, CA7.12, CA7.32, P19	Moderate	DTU level	Moderate	Data amenable to numerical taxonomic analysis	Requires live strains Strain profiles may not be stable due to expansion/contraction of tandem repeats Prone to convergence b/w unrelated strains	[144,154,155,309]
DNA fingerprinting	Analysis of variability in nuclear minisatellites by restriction digestion and probe hybridization	33.15	Low	Intra-lineage	Low	Hypervariable markers Can produce strain-specific profiles	Requires live strains Reproducibility issues	[156]
LSSP-PCR	Analysis of size polymorphisms in mHVR amplified by LSSPs	mHVR	Low	DTU level	Low	Highly sensitive Can be used to detect *Trypanosoma cruzi* in infected tissues without parasite isolation	Reproducibility issues Potential problems of contamination due to very high copy number kDNA signatures may vary with DNA template amount and quality	[310–312]
SSCP	Analysis of size polymorphisms in multicopy gene fragments	SL-IR, 24Sα rRNA, 18S rRNA, cruzipain, P7-P8	Moderate	DTU level	Low	Requires limited technical expertise	Requires live strains DTU assignment based on presence/absence of amplicons; insensitive to potential mutations in novel strains Unknown intra-strain copy homology	[25,313,314]
PCR product size polymorphism	Analysis of size polymorphisms in multicopy gene fragments	SL-IR, 24Sα rRNA, 18S rRNA, A10	High	DTU level	Low	Can be performed directly on field samples Requires limited technical expertise	DTU assignment based on presence/absence of amplicons; insensitive to potential mutations in novel strains Unknown intra-strain copy homology	[41–43]
PCR-RFLP	RFLP analysis of multicopy gene fragments	HSP60, GPI, COII, GP72, 1F8, histone H3, ITS, TcSC5D	High	DTU level	Moderate	Can be performed directly on field samples Requires limited technical expertise	DTU assignment based on presence/absence of SNPs; insensitive to potential mutations in novel strains	[94,158,315]
Nucleotide sequencing: nuclear loci (nMLST)	SNP analysis of nuclear housekeeping gene fragments	TcMSH2, DHFR-TS, TR, LYT1, Met-II, Met-III, TcAPX, TcGPX, TcMPX, HMCOAR, PDH, GTP, STTP2, RHO1, GPI, SODA, SODB, LAP	High	DTU level (intra-lineage)	High	Data amenable to MLST analysis Data highly reproducible, portable and transferable b/w laboratories	Requires live strains Level of intra-lineage resolution dependent upon analysis of multiple loci	[60,61,298,316]
Nucleotide sequencing: maxicircle loci (mtMLST)	SNP analysis of mitochondrial gene fragments	12S rRNA, 9S rRNA, Cytb, MURF1, ND1, COII, ND4, ND5, ND7	High	[DTU-level] Intra-lineage	High	Data amenable to MLST analysis Data highly reproducible, portable and transferable b/w laboratories	Requires live strains Potential phylogenetic incongruence with nuclear loci Identifies three maxicircle classes (TcI, TcII and TcIII–VI); not specific to all six DTUs	[54,317,318]
Nucleotide sequencing: minicircle regions	SNP analysis of mHVR	mHVR	High	DTU level Intra-lineage	High	Hypervariable markers Can produce strain-specific profiles	Strain profile may not be DTU specific; minor sequence classes shared b/w DTUs	[137,138]
FFLB	Analysis of size polymorphisms in multicopy gene fragments	28Sα rRNA, 18S rRNA	High	DTU level	High	Can be performed directly on field samples	Unable to differentiate hybrid lineages (TcV and TcVI)	[319]
HRM	Analysis of amplicon melting temperatures generated by real-time PCR	SL-IR, 24Sα rRNA	High	DTU level	Moderate	Data rapidly generated in real time	Requires live strains Difficult to standardize b/w laboratories Requires specialized laboratory infrastructure	[320]
MLMT	Analysis of size polymorphisms of microsatellite repeat regions	10101(CA)_a_, 11283(TA)_b_, 7093(TA)_b_, TcUn4, mclf10, 10187(CA)(TA), 6855(TA)(GA), 10359(CA), 8741(TA), 10187(TTA), 7093(TA)_c_	Moderate	DTU level Intra-lineage	High	Neutrally evolving, co-dominant, hypervariable markers Can produce strain-specific MLGs	Requires live strains Prone to homoplasy Data interpretation highly subjective	[53,70,78,89,133,134,157,321]
Amplicon sequencing	Analysis of millions of sequencing reads generated by Illumina deep sequencing	TcGP63, ND5	High	DTU level Intra-lineage Parasite multiclonality	Very high	Can detect intra-host parasite multiclonality and genetic diversity	Requires live strains Prone to loss of clonal diversity from parasite isolation Requires bioinformatics expertise, computational infrastructure and comparatively high cost reagents	[289]

aCSDI: Absolute chromosomal size difference index; DTU: Discrete typing unit; FFLB: Fluorescent fragment length barcoding; GPI: Glucose-6-phosphate isomerase; HRM: High-resolution melting; HSP60: Heat shock protein 60; kDNA: Kinetoplast DNA; LSSP: Low stringency single specific primer; mHVR: Minicircle hypervariable region; MLEE: Multilocus enzyme electrophoresis; MLG: Multilocus genotype; MLMT: Multilocus microsatellite typing; mtMLST: Maxicircle multilocus sequence typing; nMLST: Nuclear multilocus sequence typing; PFGE: Pulsed-field gel electrophoresis; RAPD: Random amplification of polymorphic DNA; RFLP: Restriction fragment length polymorphism; SL-IR: Spliced-leader intergenic region; SNP: Single nucleotide polymorphism; SSCP: Single-stranded DNA conformation polymorphism.

Direct clinical genotyping is currently based on size polymorphisms in multi-copy genetic markers, including the nuclear spliced-leader intergenic region, 24α rDNA [41], 18S rDNA [46], A10 [135] and kinetoplast hypervariable minicircle sequences [136–139] (for more detailed descriptions of historical genotyping techniques, see [55,140,141]). One major confounder associated with the use of any multi-copy gene is the level of intra-clone copy number and position homology to ensure comparability between strains. Genome size [142,143], karyotype [144–148] and chromosomal arrangements of tandem repeat regions [149,150] are known to differ widely between natural *T. cruzi* strains and even biological clones derived from the same population. Similar caveats affect minicircle-based genotyping, which vary in copy number and complement between major DTUs [136,151], are susceptible to contamination [152] and whose profiles are highly sensitive to minor changes in reaction conditions, raising issues of reproducibility [121,153]. With many of these methods, strain DTU assignment is dependent on absence of PCR products/restriction fragment bands, which can also result from novel variation in as yet untested strains; a typing methodology is only as ‘good’ as the panel of reference strains used to validate it.

Additional genotyping options are available for axenic parasite cultures, including karyotyping [154,155], DNA fingerprinting [156] and microsatellite analyses [53,70,78,89,157]. To date, no single, widely validated genetic marker affords complete, unequivocal DTU resolution [158], and reliance on only one target is inadvisable given the potential confounding influence of genetic exchange [57]. The availability of reference whole genome sequences [159–162] has reinvigorated interest in exploring the relevance of *T. cruzi* genetic diversity to clinical outcomes of Chagas disease. However, comparative genomics of representative *T. cruzi* field isolates is not yet a reality, as is the case with other more experimentally tractable trypanosomatid species [163–165].

## Methods for the systematic literature review

To date, few correlates of chronic disease progression and clinical manifestations have been identified, although both host and parasite genetics are presumed to be involved [140,166,167]. Herein, the authors systematically review the literature, given our current understanding of *T. cruzi* genetic diversity, to re-evaluate the evidence for any association between parasite genotype and clinical outcome, risk of congenital transmission or reactivation, and orally transmitted outbreaks.

Independent queries of the literature were performed using the electronic databases MEDLINE/PubMed, Web of Science v5.15, EMBASE and Scopus, with no restrictions to language or calendar date. To retrieve chronic patient studies, the following search terms were used: ‘chronic’ or ‘patients’ AND ‘Chagas disease’ or ‘cruzi’ AND ‘DTU’ or ‘DTUs’ or ‘lineage’ or ‘lineages’ or ‘genotype’ or ‘genotypes’ or ‘genotyping’. To retrieve congenital studies, the following search terms were used: ‘congenital’ or ‘maternal’ or ‘neonate’ AND ‘Chagas disease’ or ‘cruzi’ AND ‘DTU’ or ‘DTUs’ or ‘lineage’ or ‘lineages’ or ‘genotype’ or ‘genotypes’ or ‘genotyping’. To retrieve reactivation studies, the following search terms were used: ‘HIV’ or ‘transplant’ or ‘reactivation’ AND ‘Chagas disease’ or ‘cruzi’ AND ‘DTU’ or ‘DTUs’ or ‘lineage’ or ‘lineages’ or ‘genotype’ or ‘genotypes’ or ‘genotyping’. To retrieve oral transmission studies, the following search terms were used: ‘oral’ or ‘acute’ AND ‘Chagas disease’ or ‘cruzi’ AND ‘DTU’ or ‘DTUs’ or ‘lineage’ or ‘lineages’ or ‘genotype’ or ‘genotypes’ or ‘genotyping’. In addition, reference lists from retrieved articles were manually checked to identify further relevant studies.

For cohorts of chronic chagasic patients, original research studies that met all of the following criteria were included: at a minimum, patients were classified as ‘acute’, ‘indeterminate’ (i.e., asymptomatic) or ‘chronic’ (i.e., symptomatic) following clinical examination; *T. cruzi* genotyping was performed on a subset of infected patients, at least to DTU level; and results were reported with reference to a recognizable *T. cruzi* nomenclature scheme, as detailed in Table 1.

For reactivation patients, original research studies that met all of the following criteria were included: patients were clinically classified as immunocompromised by confirmation of HIV co-infection or following organ transplantation; *T. cruzi* genotyping was performed on a subset of infected patients, at least to DTU level; and results were reported with reference to a recognizable *T. cruzi* nomenclature scheme (Table 1).

For congenital patients, original research studies that met all of the following criteria were included: neonatal *T. cruzi* infection was established at birth, or shortly thereafter, but prior to early childhood to be considered ‘congenitally infected’; *T. cruzi* genotyping was performed on a subset of infected neonates and/or mothers, at least to DTU level; and results were reported with reference to a recognizable *T. cruzi* nomenclature scheme (Table 1). An additional criterion (*T. cruzi* genotyping performed on matched mother–infant samples) was dropped when the initial literature search indicated that it would have excluded all but three articles with a total of 23 mother–infant pairs [135,168,169].

For oral outbreaks, original research studies that met all of the following criteria were included: oral transmission was confirmed on the basis of epidemiological indicators (e.g., familial clustering), incrimination of the contaminated food source, clinical presentation (severe acute morbidity and/or mortality) or exclusion of local vector-borne transmission; *T. cruzi* genotyping was performed on a subset of infected patients, at least to DTU level; and results were reported with reference to a recognizable *T. cruzi* nomenclature scheme (Table 1).

Exclusion criteria included articles in languages other than English or Spanish, unpublished reports (including dissertations or conference abstracts), papers presenting solely animal data, book chapters, prospective study protocols and review articles. Patient case studies with sample size <5 were excluded from the analysis of chronic chagasic patients. Because the literature for congenital, reactivation and oral transmission is sparse by comparison, we included all publications in these categories that provided genotyping data from human-derived specimens within a recognizable *T. cruzi* nomenclature scheme.

## Chronic Chagas disease

The earliest evidence that chronic Chagas disease manifestations may differ according to parasite strain came from reports of geographical variation, in which the rarity of megasyndromes in Venezuela compared to central and eastern Brazil was circumstantially linked to radical genetic differences between *T. cruzi* zymodemes (Table 4) [16,17]. Based on the electrophoretic mobilities of 6 [36] to 18 isoenzymes [170], ZI (TcI) was the principal zymodeme identified in chronic patients (19/19) and domestic (13/13) and sylvatic vectors and mammals (18/20) in Venezuela. By comparison, in central and eastern Brazil, ZII (TcII) was the most prevalent zymodeme in acute and chronic patients (98/99) and domestic transmission cycles (9/9), but not sylvatic reservoirs (ZI [TcI]: 23/25; ZIII [TcIV]: 2/25). This dichotomy between principal parasite types was reinforced by parallel observations from other regions of Brazil [37,129,171,172]. In Belém, north Brazil, ZI (TcI) and ZIII (TcIV) were incriminated in oral outbreaks and both were found circulating among acute cases (3/7 and 4/7, respectively) and the sylvatic environment (ZI [TcI]: 106/118; ZIII [TcIV]: 6/118; ZIII ASAT [TcIII]: 6/118) [16], while to the south-east, ZII (TcII) was associated with chronic human Chagas disease in São Felipe, Bahia [37]. In neighboring parts of Goiás, Bahia and Minas Gerais, comparable proportions of ZI (TcI) and ZII (TcII) were isolated from acute cases, with similar clinical courses, but only ZII was identified in chronic patients presenting a range of cardiac and digestive symptoms [129,173].

**Table 4.** **Summary of clinical Chagas disease publications, which included genotyping to discrete typing unit level by multilocus enzyme electrophoresis: years 1980–2002.**

**Study region, country, year**	**Sample size and clinical classification^†^**	**Type of clinical sample **	**Method of clinical classification **	**Patient DTU^‡^**	**Genotyping methodology**	**Ref.**
Endemic regions, Argentina (1992)^§^	15 acute 12 IND 5 CCM	Parasite cultures from xenodiagnoses	ND	Acute: 7 TcI (Z2, Z12), 8 TcV (Z1) IND: 2 TcI (Z2, Z12), 10 (83%) TcV (Z1) CCM: 3 (60%) TcI (Z2, Z12), 1 TcII (Z11), 1 TcV (Z1)	MLEE (6 loci) [180]	[178]
Endemic regions, Argentina (1993)^§^	15 acute 14 IND 8 CCM	Parasite cultures from xenodiagnoses	ND	Acute: 7 TcI (Z2, Z12), 8 TcV (Z1) IND: 1 TcI (Z2, Z12), 13 (93%) TcV (Z1) CCM: 6 (75%) TcI (Z2, Z12), 1 TcII (Z11), 1 TcV (Z1)	MLEE (10 loci) [180]	[295]
Endemic regions, Argentina (1996)	7 acute 35 IND 19 CCM 1 CCM-DIG	Parasite cultures from xenodiagnoses	Chest x-ray ECG [322]	Acute: 4 TcI (Z2, Z12), 3 TcV (Z1) IND: 4 TcI (Z2, Z12), 1 TcII (Z11), 30 (86%) TcV (Z1) CCM: 10 (53%) TcI (Z2, Z12), 2 TcII (Z11), 7 TcV (Z1) CCM-DIG: 1 TcII (Z11)	MLEE (6 loci) [180]	[177]
Goiás, Bahia, Minas Gerais, Brazil (1986)	25 acute 1 IND 1 CCM 7 ME 3 CCM-DIG	Parasite cultures from xenodiagnoses	Chest x-ray ECG Barium swallow Barium enema [172,323]	Acute: 12 TcI (ZI), 13 (52%) TcII (ZII) IND (1), CCM (1), ME (7), CCM-DIG (3): all TcII (ZII) (100%)	MLEE (15 loci) [128]	[129]
Amazonian, central and eastern, Brazil; Venezuela (1981)	Venezuela: 11 IND 8 CCM Amazonian Brazil: 7 acute CE Brazil: 99 acute and chronic	Patient cultures from xenodiagnoses, hemoculturing or animal inoculations	ND	Venezuela: IND (11), CCM (8): 19 (100%) TcI (ZI) Amazonian Brazil: Acute: 3 TcI (ZI), 4 TcIV (ZIII) CE Brazil: Acute and chronic: 1 TcI1 (ZI), 98 (99%) TcII (ZII)	MLEE (6–18 loci) [36,70]	[16]
Bahia, Brazil (1980)	22 acute 12 IND 21 CCM 1 ME/MC 1 congenital	Parasite cultures from xenodiagnoses and hemoculturing	ND	Acute: 11 TcI (ZI), 11 TcII (ZII) IND: 12 TcII (ZII) (100%) CCM: 1 TcI (ZI), 20 TcII (ZII) (95%) ME/MC: 1 TcII (ZII) Congenital: 1 TcII (ZII)	MLEE (6 loci) [36]	[173]
Cochabamba, Potosí, Santa Cruz, Sucre, Tarija, Tupiza, Valle Grande, Yungas, Bolivia (1989)	11 IND 10 CCM 6 MC 3 CCM-DIG	Parasite cultures from xenodiagnosis	ECG Barium swallow Barium enema	IND: 6 TcI (Clonet 7, 19, 20), 5 TcV (39) CCM: 6 TcI (7, 19, 20), 4 TcV (39) MC: 5 TcI (7, 19, 20), 1 TcV (39) CCM-DIG: 3 mixed TcI + V (7/19/20 + 39)	MLEE (12 loci) [181]	[174]
Regions II, III and IV, Chile (1987)	53 IND 49 CCM	Parasite cultures from xenodiagnosis	ECG	IND: 7 TcII (Brazilian ZII), 46 (87%) TcV (Bolivian ZII) CCM: 2 TcI (ZI), 9 TcII (Brazilian ZII), 38 (78%) TcV (Bolivian ZII)	MLEE (8 loci) [36]	[176]
Regions II, III and IV, Chile (1987)	63 IND 36 CCM	Parasite cultures from xenodiagnosis	ECG	IND: 8 TcII (Brazilian ZII), 55 (87%) TcV (Bolivian ZII) CCM: 2 TcI (ZI), 8 TcII (Brazilian ZII), 26 (72%) TcV (Bolivian ZII)	MLEE (5 loci) [36]	[175]
El Oro, Zamora Chinchipe, Ecuador (2002)	3 IND 1 CCM 4 CCM-DIG 2 DIG	Parasite cultures	ECG Chest x-ray Barium swallow Barium enema	IND: 1 TcI (ZI), 1 TcIV (ZIII), 1 TcV (Bolivian ZII) CCM: 1 TcV (Bolivian ZII) CCM-DIG: 1 TcI (ZI), 2 TcIV (ZIII), 1 TcV (Bolivian ZII) DIG: 2 TcV (Bolivian ZII)	MLEE (19 loci) [324]	[86]
Pr. Hayes, San Pedro, Central, Caaguazú, Cordillera, Paraguari, Paraguay (2001)	8 acute 2 IND 1 CCM 1 congenital	Patient cultures from xenodiagnoses, hemoculturing or animal inoculations	ND	Acute: 4 TcII (Brazilian ZII), 4 TcVI (Paraguayan ZII) IND: 1 TcII (Brazilian ZII), 1 TcV (Bolivian ZII) CCM: 1 TcII (Brazilian ZII) Congenital: 1 TcV (Bolivian ZII)	MLEE (15 loci) [325]	[326]

^†^Refers to number of clinical samples genotyped to DTU-level only, not total cohort size. ^‡^Genotypes have also been listed according to current DTU nomenclature [31] with original classification in parentheses. §Unspecified degree of overlap in patient populations from these two studies. CCM: Chagas cardiomyopathy; DIG: Digestive, target organ unspecified; DTU: Discrete typing unit; IND: indeterminate; ND: Not described; MC: Megacolon; ME: Megaesophagus.

Further south, most clinical reports supported the principal involvement of Bolivian ZII (TcV) in chronic Chagas disease [174–176]. In northern Chile, the majority of patients, regardless of symptom status, were infected with Bolivian ZII (TcV) (64/85 and 101/116 CCM and IND patients, respectively) [175,176]. However, one study in Bolivia detected TcI (clonet 7, 19 or 20) and TcV (clonet 39) in almost equal proportions (17/27 and 10/27, respectively) from both IND and symptomatic chronic individuals [174]. The latter study was one of the first to describe a number of mixed infections (as either a mixed isoenzyme profile or different isoenzyme profiles from sequential parasite samples from an individual patient), including three patients with TcI/TcV (clonets 7, 19 or 20 and 39) co-infections presenting both cardiac and digestive abnormalities [174]. Finally, in Argentina, stronger evidence of a link between parasite genetics and progression to symptomatic disease was reported, with TcV prevailing in the IND form (30/35 [86%]) and CCM significantly more associated with TcI (10/14 [71%]) than TcV (7/37 [19%]) [177].

To date, these remain some of the largest cross-sectional studies in which infecting *T. cruzi* genotype was directly examined in conjunction with patient clinical data (Table 4). Investigators noted that digestive Chagas disease was frequent in the Southern Cone, coinciding with the absence of TcI and preponderance of TcII/V, and drew a contrast with the rarity of digestive disease and predominance of TcI further north [16]. However, in these studies, very little genotypic data were obtained for digestive patients (n = 16; 5 TcI, 8 TcII and 3 TcV) compared to those with CCM (n = 159; 38 TcI, 43 TcII and 78 TcV) or without symptomatic disease (n = 217; 25 TcI, 30 TcII, 1 TcIV and 161 TcV) (Table 4). Furthermore, some of the aforementioned experimental design and biological limitations must be acknowledged alongside these observations. In all of these early reports, MLEE was performed using lysate prepared from parasites isolated through a combination of hemoculture, xendiagnosis and/or inoculation into animals [129,173] and in each study, positive hemocultures were obtained for less than one-third of patients (107/391 in Chile [176] and 14/111 in Brazil [129]). Mixed infections were identified by a handful of investigators who undertook biological cloning of strains or sequential sampling [174,178], but were not routinely investigated. At the time, research groups were using separate MLEE protocols (although latterly determined to be comparable [179]), different and varying standards of clinical classification (some omitting any GI examination altogether) [36,128,170,180,181], and finally confusing and conflicting *T. cruzi* strain nomenclatures [1,16,42,43,129,177,174,176], hindering any prospective meta-analysis across endemic regions.

With improved molecular techniques and the advent of direct genotyping from clinical specimens, current evidence suggests that parasite strains detected in peripheral blood from patients with or without morbidity reflect the principal lineage circulating in the local domestic cycle (Table 5). However, it should also be noted that due to differential strain tropisms, bloodstream parasites are not necessarily the same genotype responsible for pathology [140]. In studies from northern Brazil, Colombia, Guatemala, Mexico and Panama, TcI predominates in both IND and CCM groups [182–187]; a minority of infections in Colombia were attributable to TcII (5/26 IND and 6/41 CCM) [183,184]. The two largest recent endeavors to compare *T. cruzi* genotypes in symptomatic versus asymptomatic Chagas disease patients were conducted in Argentina (n = 172) [188] and Bolivia (n = 132) [189] and support the association of chronic infection, independent of symptom status, with TcII/V/VI (149/149 IND, 98/98 CCM, 5/5 CCM-megacolon (MC) and 40/44 MC). Despite the use of more sensitive molecular genotyping techniques, results for approximately 30–50% of specimens were missing due to low parasite load in peripheral blood. Additional smaller studies also corroborate these observations with domestic genotypes detected in both patients’ blood [190,191] and cardiac, esophagus and colon tissue specimens [192,193].

**Table 5.** **Summary of clinical Chagas disease publications, which included genotyping to discrete typing unit level: years 2005–2014.**

**Study region, country, year**	**Sample size and clinical classification^†^**	**Type of clinical sample **	**Clinical classification **	**Patient DTU^‡^**	**Genotyping methodology**	**Ref.**
Endemic and non-endemic regions Argentina (2012)	95 IND 69 CCM (blood) 8 CCM (heart tissue)^§^	Venous blood Tissue: explanted heart, endomyocardial	ND	IND: 1 TcII/VI, 34 TcII/V/VI (36%), 54 TcV (57%), 1 TcVI, 5 TcV or TcV + II/VI CCM (blood): 50 (72%) TcII/V/VI, 16 (23%) TcV, 1 TcII/VI, 2 TcV or TcV + TcII/VI CCM (tissue): 3 (38%) TcI, 2 (25%) TcII/VI, 2 (25%) TcII/V/VI, 1 TcVI	*Nuclear* SL-IR 24Sα rDNA A10 (all) [135]	[188]
Cochabamba, Bolivia (2006)	18 MC	Colon tissue	ND	MC: 2 TcII, 16 TcV (89%)	*kDNA* Southern blots [137,327]	[192]
Santa Cruz, Bolivia (2010)	54 IND 29 CCM 5 CCM-MC 15 MC 29 MC-UK cardiac status	Venous blood	ECG Colon x-ray Barium enema	IND: 1 TcII, 9 (17%) TcI/V, 44 (81%) TcV CCM: 2 TcII, 6 (21%) TcI/V, 20 (69%) TcV, 1 TcVI CCM-MC: 1 TcII/V, 4 TcV MC: 3 TcI/V, 1 TcII/V, 11 (73%) TcV MC-UK: 4 TcI, 3 TcII, 4 TcI/V, 1 TcI/II/V, 17 TcV (59%)	*kDNA* Southern blots [327]	[189]
Manaus, Brazil (2014)	11 IND 2 CCM	Xenodiagnoses feces	ND	IND: 11 (100%) TcI CCM: 2 (100%) TcI	*Nuclear* SL-IR [41] *kDNA* COII [54]	[182]
Goiás, Bahia, Minas Gerais, Brazil (2009)	27 IND 17 CCM	Parasite cultures from hemoculturing	ECG Chest x-ray Echocardiogram	IND: 26 TcII (96%), 1 TcIII CCM: 16 TcII (94%), 1 TcV/VI	*Nuclear* SL-IR 24Sα rDNA [135] Microsatellites (9 loci) [102] *kDNA* COII [54]	[190]
Minas Gerais, Brazil (2006)	8 IND 19 CCM 10 ME 2 MC 18 CCM-ME 3 CCM-MC 10 CCM-MC-ME	Parasite cultures from hemoculturing	ECG Barium swallow Barium enema	IND: 8 *Trypanosoma cruzi* II CCM: 19 *T. cruzi* II ME: 10 *T. cruzi* II MC: 2 *T. cruzi* II CCM-ME: 18 *T. cruzi* II CCM-MC: 3 *T. cruzi* II CCM-MC-ME: 10 *T. cruzi* II	*Nuclear* 24Sα rDNA [41]	[191]
Goiás, Bahia, Minas Gerais, Brazil (2005)	11 CCM 16 ME 1 MC	Tissues: heart, esophagus, colon	ND	CCM: 11 *T. cruzi* II ME: 16 *T. cruzi* II MC: 1 *T. cruzi* II	*Nuclear* 24Sα rDNA [193]	[193]
IV, V and Metropolitan regions, Chile (2009)^¶^	17 IND 13 CCM	Venous blood (XD results excluded)^‡^	ECG Chest x-ray Echocardiogram	IND: 1 TcII, 1 TcI + II, 2 TcII + hybrid, 13 (76%) TcI + II + hybrid CCM: 1 TcI, 1 TcII, 1 TcII + hybrid, 10 (77%) TcI + II + hybrid	*kDNA* Southern blots [327]	[197]
IV, V and Metropolitan regions, Chile (2010)^¶^	33 IND 28 CCM	Venous blood (XD results excluded) ^‡^	ECG Chest x-ray Echocardiogram	IND: 26 (79%) TcI, 5 TcII, 1 TcIII, 1 TcV\VI CCM: 16 (57%) TcI, 6 TcII, 6 TcIII	*Nuclear* Microsatellites (3 loci)	[198]
IV region, Chile (2013)^¶^	28 IND 24 CCM	Venous blood^‡^	ND	IND: 24 (86%) TcI, 3 TcII, 1 TcIII CCM: 17 (71%) TcI, 6 TcII, 1 TcIII	*Nuclear* Microsatellites (3 loci) [133]	[199]
Santander, Colombia (2010)	28 IND 31 CCM	Venous blood	ECG Holter echocardiogram	IND: 21 (75%) TcI, 5 TcII, 2 TcI + II CCM: 27 (87%) TcI, 4 TcII	*Nuclear* SL-IR 24Sα rDNA [41] kDNA COII [54]	[183]
Santander, Colombia (2011)	10 CCM	Cardiac tissue (autopsy)	Pathology only	CCM: 8 (80%) TcI, 2 TcII	*Nuclear* SL-IR [41]	[184]
Endemic regions, Guatemala and Mexico (2005)	Guatemala: 3 Acute 1 IND Mexico: 1 Acute 4 IND 7 CCM	Parasite cultures from hemoculturing	ECG Chest x-ray	Acute: 4 (100%) TcI IND: 5 (100%) TcI CCM: 7 (100%) TcI	*Nuclear* SL-IR [41]	[185]
Endemic regions, Mexico (2013)	3 Acute 11 IND 6 CCM	Parasite cultures from hemoculturing	ND	Acute: 3 (100%) TcI IND: 11 (100%) TcI CCM: 6 (100%) TcI	*Nuclear* SL-IR [41]	[186]
Endemic regions, Panama (2006)	5 acute 7 IND 11 CCM	Xenodiagnoses and hemoculturing	ND	Acute: 5 (100%) TcI IND: 7 (100%) TcI CCM: 11 (100%) TcI	*Nuclear* SL-IR [41]	[187]

^†^Patients classified as indeterminate if no barium studies are performed, patients with megacolon by barium studies but no ECG are classified as MC-UK cardiac status. ^‡^XD omitted as specimens could not be matched to patients. ^§^Same heart explants reported in [104]. ^¶^Participants in follow-up after allopurinol clinical trial. Unspecified degree of overlap in patient populations in these three studies. CCM: Chagas cardiomyopathy; DTU: Discrete typing unit; IND: Indeterminate; kDNA: Kinetoplast DNA; ME: Megaesophagus; MC: Megacolon; ND: Not described; SL-IR: Spliced-leader intergenic region; UK: Unknown status; XD: Xenodiagnostic data.

One recent method, developed to circumvent some of these technical limitations, is to adopt an indirect approach, exploiting serological detection of antibodies produced in response to DTU-specific *T. cruzi* antigens, with the advantage of potentially revealing both historical and contemporary infecting parasite lineages. Consistent with genotypic data from the same area, serosurveys in southern endemic regions using recombinant peptides directed at trypomastigote small surface antigen indicate pervasive infection with TcII/V/VI [194,195] and even a putative association of ECG abnormalities with seroreactivity to this particular protein [196].

Lastly, on a more cautionary note, three publications from Chile report conflicting genotypic data from patients in longitudinal follow-up after a clinical trial of allopurinol and itraconazole (Table 5) [197–199]. In the first publication, using kDNA Southern blots, more than 75% of the results in both groups were described as ‘TcI + TcII + hybrid’ [197], perhaps reflecting cross-reactivity between minicircle hybridization probes [200]. Subsequent papers presenting nuclear microsatellite genotyping from patients from the same clinical trial, with unspecified degrees of overlap, demonstrated a predominance of TcI (79% of 33 IND and 57% of 28 CCM in [198]; 86% of 28 IND and 71% of 24 CCM in [199]).

## Chagas disease reactivation

Reactivation of Chagas disease may occur in infected individuals who become immunocompromised through immunosuppressive treatment (e.g., transplant recipients) or co-infection with HIV [11,201]. Reactivation is characterized by increased parasite multiplication, a return to microscopically detectable parasitemia levels and symptoms more typical of acute *T. cruzi* infection, including meningoencephalitis, acute myocarditis and skin chagomas [201]. High parasite loads lead to more frequent strain identification in immunocompromised than immunocompetent patients [188]. In addition, the loss of immunological control may allow parasites previously sequestered in deep tissues to replicate and return to the circulation. Thus, patients with reactivation arguably provide the best *in vivo* indicator of the complexity of natural infections in humans [102–104].

Compared to results from immunocompetent individuals, the detection of patent mixed infections is striking in some immunocompromised patients, with multiple genotypes in a single specimen or different genotypes in tissue compared to blood (Table 6). TcI, hypothesized to have cardiac muscle tropism, demonstrated contrasting distributions in tissue versus blood in immunocompetent patients (38% in cardiac tissue vs 0% in blood) [188], but was detected at equal frequencies in tissue and blood from cardiac transplant patients with reactivation (31% in tissue vs 33% in blood) (Table 6) [188]. This result may reflect unmasking of mixed infections that were below the level of detection prior to reactivation.

**Table 6.** **Summary of clinical publications which included genotyping to discrete typing unit level in immunosuppressed patients with *Trypanosoma cruzi* reactivation.**

**Study region, country, year**	**Sample size and clinical classification**	**Type of clinical sample **	**Patient DTU**	**Genotyping methodology**	**Ref.**
Buenos Aires, Argentina (2005)	1 HIV-*Trypanosoma cruzi* co-infected patient with CNS reactivation	Venous blood Brain biopsy	Blood: TcV Brain: TcII/VI	Nuclear SL-IR 24Sα rDNA A10 [135]	[328]
Buenos Aires, Argentina (2008)	1 HIV-*T. cruzi* co-infected patient with CNS reactivation	Venous blood CSF	Blood: TcV/VI CSF: TcI	Nuclear SL-IR, 24Sα rDNA A10 [135]	[103]
Buenos Aires, Argentina (2009)	10 HIV-*T. cruzi* co-infected patients with CNS reactivation	Different combinations of blood, CSF, brain tissue	4 Pts (8, 9, 13, 17): blood TcV Pt10: blood TcV, brain TcII Pt11: blood TcI + TcV, CSF TcI Pt12: blood UD, brain TcV Pt14: blood TcV, CSF UD Pt15: CSF TcII/V/VI Pt16: blood UD	Nuclear SL-IR 24Sα rDNA A10 [135]	[329]
Buenos Aires, Argentina (2010)	6 patients post-heart transplantation with reactivation in heart (1), skin (4), and heart and skin (1)	Blood, heart, skin	4 patients with blood–skin: 2 TcI in both, 1 TcV in both, 1 TcI in blood, TcII/VI in skin 1 with blood-heart: TcV in both 1 with blood–heart–skin: TcVI in all	Nuclear SL-IR 24Sα rDNA A10 [135]	[104]
Argentina (2012)	25 patients with reactivation due to HIV or post-cardiac transplant^†^	Blood, heart, skin, brain	Blood: 7 TcI, 1 TcII, 7 TcV, 1 TcII/VI, 4 TcV or V + II/VI, 1 TcVI Tissue: 4 TcI, 2 TcII, 2 TcV, 2 TcII/VI, 1 TcV or V + II/VI, 2 TcVI	Nuclear SL-IR 24Sα rDNA A10 [135]	[188]
Argentina (2013)	5 recipients of contaminated organ transplants	Blood	Pt1A (lung): TcV or TcV + TcVI 3 Pts (1B, 2A, 3A; liver): TcV or TcV + TcVI Pt4A (kidney): TcV or TcV + TcVI	Nuclear SL-IR 24Sα rDNA A10 [135]	[330]
Brazil (2002)	1 HIV-*T. cruzi* co-infected patient with CNS reactivation	Blood, CSF	Blood: *T. cruzi* II CSF: *T. cruzi* II	Nuclear 24Sα rDNA [41]	[331]
Brazil (1999)	28 HIV-*T. cruzi* co-infected patients 18 *T.* cruzi infected patients	Parasite cultures from xenodiagnosis	HIV+: 25 TcII (Clonet 30, 32), 1 TcV (39), 2 TcVI (43) HIV−: 17 TcII, 1 TcV	Nuclear MLEE (21 loci) [324] RAPD [39]	[332]
Colombia (2014)	1 HIV-*T. cruzi* co-infected patient with CNS reactivation	Post-mortem heart and brain tissue	Mixed TcI sylvatic/TcI_DOM_ in heart, only TcI sylvatic in brain	Nuclear SL-IR, 24Sα rDNA A10 [135]	[333]

^†^Unspecified degree of overlap with [104,328,329]. CSF: Cerebral spinal fluid; DTU: Discrete typing unit; MLEE: Multilocus enzyme electrophoresis; RAPD: Random amplification of polymorphic DNA; Pt: Patient; SL-IR: Spliced-leader intergenic region; UD: Undetectable.

Current data are insufficient to evaluate any association between strain and clinical symptoms or risk of reactivation. Analyses need to be designed to account for known risk factors for reactivation (severity of immunocompromise and specific immunosuppressive regimens) [201]. The most powerful design is a longitudinal approach, for example, in end-stage CCM patients evaluated for heart transplantation, combined with parasitological monitoring following surgery. However, pre- and post-immunosuppression comparisons are difficult, given that genotyping from blood of immunocompetent patients is limited by low peripheral parasitemia [104].

## Congenital Chagas disease transmission

With improved vector control, congenital transmission has become proportionately more important among chronically infected populations, estimated to account for 22% of new *T. cruzi* cases in 2015 [1]. Even if vector-borne transmission were interrupted today, infected girls and women will continue to transmit the infection to their children, sustaining the cycle across generations in the absence of the vector [202].

Congenital *T. cruzi* infection is most often clinically silent, but can cause a spectrum of presentations, including low birth weight, prematurity and low Apgar scores to meningoencephalitis, hepatosplenomegaly, anemia, thrombocytopenia and respiratory distress syndrome [203–205]. Higher morbidity and mortality rates were described in the 1980s and 1990s compared to more recent cohort data [204,206]. Clinically severe congenital infection is reported to be associated with higher levels of neonatal parasitemia than less-severe or asymptomatic disease [207]. Congenital Chagas disease is also assumed to carry the same risk of chronic cardiac and/or GI manifestations as vector-borne infections.

Since the earliest descriptions of congenital *T. cruzi* infection [208–210], researchers have struggled to explain why vertical transmission is restricted to only a small proportion of infected mothers. Congenital transmission rates are highly variable both within and between endemic areas, ranging from 4.4 to 11.3% in Argentina [211–214], from 3.4 to 17.1% in Bolivia [204,205,215,216], from 0.2 to 5.2% in Brazil [217,218], from 2.5 to 11.1% in Chile [219–221] and from 5.6 to 10% in Paraguay [222,223]. Factors now known to be associated with higher risk of congenital transmission include younger maternal age (presumed to reflect more recent infection) [204], maternal and neonatal immunological responses [224,225], higher maternal parasitemia [168,205,214], and HIV and other immunodeficiencies [226,227]; the evidence for any influence of *T. cruzi* genetic diversity is more equivocal (Table 7). The majority of congenital genotyping studies have been performed in southern endemic areas, particularly Argentina, Bolivia and Chile, and in general, mirror the distribution of TcII/V/VI lineages observed among local chronic adult populations; additional studies are needed from regions of domestic TcI transmission in northern South and Central America [228,229].

**Table 7.** **Summary of congenital Chagas disease publications, which included genotyping to discrete typing unit level.**

**Region, country, year**	**Sample size^†^**	**Type of clinical sample **	**Maternal *Trypanosoma cruzi* DTU**	**Neonate *T. cruzi* DTU**	**Genotyping/serotyping methodology**	**Ref.**
Buenos Aires, Argentina (2013)	19 neonates	Parasite hemocultures from neonatal blood	ND	TcI (n = 1) TcV (n = 18)	Nuclear SL-IR 24Sα rDNA [41]	[214]
Endemic and non-endemic regions, Argentina (2012)	51 neonates	Neonatal cord or venous blood	ND	TcV (n = 18) TcII/V/VI (n = 6) TcV or mixed V + II/VI (n = 27)	Nuclear SL-IR 24Sα rDNA A10 [135]	[188]
Buenos Aires, Argentina (2011)	35 mothers: 20 Argentinian, 6 Bolivian, 9 Paraguayan	Maternal sera (n = 26) Maternal blood (n = 9) Placenta (n = 2)	TcI (n = 1 Argentinan) TcII/V/VI (n = 26; 17 Argentinian, 3 Bolivian and 6 Paraguayan)	ND	Nuclear SL-IR [135] kDNA Minicircle RFLPs [234] Serology TSSA-I/II [195]	[234]
Reconquista, Argentina (2010)	14 neonates	Neonatal venous blood	ND	TcV (n = 7) TcVI (n = 1) TcII + TcV (n = 1) TcII + TcVI (n = 1) TcV + TcVI (n = 4)	kDNA Southern blots [327] mHVR-specific PCR [138]	[231]
Salta, Argentina (2009)	18 neonates	Parasite hemocultures from neonatal cord and venous blood	ND	TcV (n = 18)	Nuclear MLEE (11 loci) [324] RAPD [45] kDNA Southern blots [139,200]	[304]
Buenos Aires, Argentina (2009)	2 neonates	Neonatal venous blood	ND	TcV (n = 2)	Nuclear SL-IR 24Sα rDNA A10 [135] kDNA Minicircle sequencing [137] LSSP-PCR [312]	[334]
Buenos Aires, Argentina (2007)	5 mothers (transmitters) 13 mothers (non-transmitters) 38 neonates	Neonatal venous blood Maternal blood	Transmitters: TcV (n = 5) Non-transmitters: TcI (n = 1) TcV (n = 12)	TcII (n = 1) TcV (n = 36) TcI + TcV/VI (n = 1 – HIV co-infected)	Nuclear SL-IR 24Sα rDNA [135] A10 Microsatellites (4 loci) [321] kDNA Minicircle RFLPs Minicircle sequencing [137]	[135]
Bahia, Brazil (1985)	7 mothers (non-transmitters) 5 neonates	Parasite hemocultures from maternal and neonatal blood	TcII (ZII; n = 7)	TcII (ZII; n = 4) TcV (Bolivian ZII; n = 1)	Nuclear MLEE (9 loci) [36]	[335]
Cochabamba and Tarija, Bolivia (2007)	15 mothers 36 neonates (matched)	Maternal blood Neonatal cord blood	TcV (n = 14) TcVI (n = 1)	TcII (n = 1) TcV (n = 34) TcVI (n = 1)	kDNA Southern blots [139,200]	[168]
Cochabamba and Tarija, Bolivia (2006)	17 mothers 41 neonates (unmatched)	Maternal blood Neonatal cord blood	TcV (n = 16) TcVI (n = 1)	TcII (n = 1) TcV (n = 39) TcVI (n = 1)	Nuclear SL-IR [41] SCAR [45] kDNA Southern blots [137] Minicircle sequencing [139]	[200]
Cochabamba, Bolivia (1995)	31 neonates	Neonatal cord or venous blood	ND	TcI (clone 20) (n = 3) TcV (clone 39) (n = 7) TcI + TcV (n = 4)	kDNA Southern blots [139]	[336]
Chile (2014)	17 neonates	Neonatal cord blood	ND	TcI (n = 2) TcII (n = 2) TcV (n = 5) TcVI (n = 1) TcI + TcII (n = 1) TcI + TcV (n = 2) TcII + TcV (n = 3) TcII + TcVI (n = 1)	kDNA Southern blots [139]	[337]
Province of Choapa, IV Region, Chile (2012)	3 mothers 3 neonates	Maternal blood Neonatal cord blood	TcI + TcII + TcV (n = 3)	TcI + TcII + TcV (n = 2) TcV (n = 1)	kDNA Southern blots [139]	[169]
Province of Choapa, IV Region, Chile (2010)	20 mothers	Maternal blood	Reported TcI and TcV single and mixed infections	ND	kDNA Southern blots [139]	[219]
Murcia, Spain (mothers from Bolivia) (2013)	9 neonates	Parasite hemocultures from neonatal venous blood	ND	TcV (n = 9)	Nuclear SL-IR [41] 24Sα rDNA 18S rDNA A10 [46]	[338]

TcII/V/VI refers reactions to markers common to all three DTUs. TcII + TcV + TcVI refers to co-infections determined by markers specific to each DTU. ^†^Refers to number of clinical samples genotype to DTU level only, not total cohort size. DTU: Discrete typing unit; kDNA: Kinetoplast DNA; LSSP: Low stringency single specific primer; mHVR: Minicircle hypervariable region; MLEE: Multilocus enzyme electrophoresis; ND: Not described; RAPD: Random amplification of polymorphic DNA; RFLP: Restriction fragment length polymorphism; SL-IR: Spliced-leader intergenic region.

A number of limitations prevent the accurate assessment of the interaction between *T. cruzi* genotype and risk of congenital transmission. Studies which examine congenital cohorts frequently perform genotyping only on maternal [219] or neonatal specimens [230], and those that incorporate both often present results from unmatched mothers and infants [200]. Even fewer studies compare parasite genotypes between mothers who transmitted to their infants and those who did not [135]. Due to the small volume of neonatal blood, most congenital genotyping is reliant on DTU-specific minicircle probes (Table 7) and some studies do not test for all lineages, but only those predicted to be found circulating locally (conventionally TcI, TcII, TcV and TcVI) [200,169,219]. Cross-reactivity between minicircle probes for closely related DTUs (TcII and TcVI) has been reported [168,200], which casts some doubt on studies detecting co-infections with these lineages [231]. Furthermore, most congenital genotyping studies are constrained by small sample sizes and suboptimal sensitivity of current conventional diagnostic methods. Microscopic examination in a single specimen fails to identify over half of infected neonates [205], and subsequent loss to follow-up is high [212,232,233], thereby routinely underestimating the rates of congenital transmission and providing only a fraction of potential parasite strains for genotyping.

Differential diagnostic sensitivities and rates of follow-up render it difficult to draw conclusions about geographical variation in congenital transmission. Multiple factors, including maternal immune response and parasite load, likely modify risk. Nevertheless, several observations support a contributory role for parasite genotype in congenital Chagas transmission risk. In Argentina, women with one congenitally infected child were significantly more likely to transmit to that child’s siblings than mothers who had not previously transmitted [234,235]. Considering the extent of *T. cruzi* genetic diversity at the intra-DTU level, it is unlikely that all representatives of a lineage would be equally permissible to vertical transmission, but it is conceivable that particular parasite clones may be better adapted for transplacental infection [235]. Animal data also support the possibility that some *T. cruzi* strains are more predisposed to vertical transmission than others [236].

To date, only one study has directly examined infected human placental tissue, detecting additional minicircle signatures not observed in matched maternal blood samples and tentatively supporting the existence of *T. cruzi* subpopulations with placental tropism [234]. Others have described parallel discordant minicircle profiles between paired maternal–neonate blood specimens, implying either the generation of novel mutations by rapid parasite multiplication during acute neonatal infection or selective transmission of parasite subpopulations [168]. In the only study to address neonatal morbidity, no significant association was found between DTU and clinical severity of congenital infection, but nearly all genotyped specimens in this study were classified as TcV [200].

In one of the aforementioned Argentinean studies, women from areas with high triatomine infestation had the lowest risk of congenital transmission compared to those with vector control (intermediate risk) or from urban areas that had never been infested (highest risk) [235]. These findings have been confirmed in the Bolivian Chaco region, where pregnant women who had resided longer in an infested house had significantly lower parasitemia and were less likely to transmit to their child, compared to those living in areas without vector infestation [237]. Sustained vector exposure and/or repeated re- or super-infection by *T. cruzi* may act as an immune booster, allowing women to maintain effective control of parasitemia, thereby decreasing their risk of congenital transmission.

## Oral *T. cruzi* transmission and outbreaks of acute Chagas disease

*T. cruzi* trypomastigotes in triatomine feces are infectious when ingested by experimental animals [238], and consumption of infected vectors or contaminated material is considered the predominant transmission modality in non-human mammals [6]. Acute *T. cruzi* infections in humans attributed to oral transmission have been reported in increasing numbers in recent decades, especially in the Brazilian Amazon [6]. Three basic scenarios are described: sporadic cases in areas with sylvatic but not domestic vectors in which the attribution is one of exclusion [6]; small rural family- or village-based clusters of acute cases traced to shared food or drink [239–242]; and rare large outbreaks, sometimes in urban areas considered to be free of vectorial transmission, with an identified common source such as contaminated fruit or sugarcane juice [243–246]. Most outbreaks are small, often affecting family groups in the Amazon region, where the palm fruits açaí and baçaba are dietary staples easily contaminated by infected triatomine vectors that live in the trees themselves [241,247].

The largest reported outbreak, attributed to locally prepared guava juice, comprised 103 infections among students and staff at a school in Caracas [244]. Orally transmitted *T. cruzi* infection appears to be associated with more severe acute morbidity and higher mortality than vector-borne infection [239,244,248]. The most frequent symptoms are fever, dyspnea, myalgias, and generalized and facial edema; ECG changes are also common. In the Caracas outbreak, 75% of 103 infected individuals were symptomatic, 66% had ECG abnormalities, 20% were hospitalized and there was one death from acute myocarditis [244,248]. Among 13 patients infected in two outbreaks associated with contaminated sugarcane juice in northeastern Brazil, 92% had ECG abnormalities, 27% had left ventricular ejection fractions below 55% and two individuals (age 9 and 16 years) died of rapidly progressive congestive heart failure [249]. Among survivors, nearly all cardiac abnormalities resolved after treatment with benznidazole. The higher proportion of symptoms and severe morbidity and clustering of infections may facilitate easier detection of acute, oral infection compared to vectorial cases, and may contribute to the predominance of oral infections in acute Chagas disease surveillance data [6].

Because of logistical constraints, few outbreaks [241,244] receive thorough epidemiological investigations, and direct incrimination of the contaminated item is infrequent. At the DTU level, *T. cruzi* genotypes identified in oral outbreaks principally reflect the predominant lineages circulating in that geographical area (Table 8). In the Caracas school outbreak, molecular typing demonstrated identical TcI strains in 3 of the 103 infected individuals and similar strains in a single *P. geniculatus* captured at the site where the implicated guava juice was prepared [244,250]. Human specimens from the outbreak in Santa Catarina in 2005 attributed to sugarcane juice were typed as TcII [40]. Small family outbreaks in the Amazon region generally yield TcIV or TcI [95,93,97,99], while rural outbreaks outside of the Brazilian Amazon (Colombia, Venezuela, French Guiana) detect TcI (sometimes identified specifically as sylvatic TcI) [240,251–253].

**Table 8.** **Summary of publications reporting oral *Trypanosoma cruzi* outbreaks which included genotyping to discrete typing unit level.**

**Outbreak location, country, year**	**Outbreak description: implicated vehicle, n infected, deaths**	**Type of clinical sample (n)**	**Patient DTU^†^**	**Genotyping/serotyping methodology**	**Ref.**
Navegantes, Santa Caterina, Brazil (2005)	Sugarcane juice, 24 infected patients, 3 deaths	Parasite hemocultures (9)	TcII (n = 9)	Nuclear MLEE (6 loci) [339] SL-IR 24Sα rDNA [135]	[243]
Coari, western Amazonia, Brazil (2007)	25 infected patients (of population 175), acute symptoms, no deaths reported	Parasite hemocultures (18)	TcIII (ZIII) (n = 18)	Nuclear SL-IR [340] kDNA COII [54]	[99]
Coari and Santa Isabel do Rio Negro, western Amazonia, Brazil (2007 and 2010)	ND	Parasite cultures from hemoculturing and xenodiagnoses (27 Coari, 15 Santa Isabel do Rio Negro)	TcIV (n = 42)	Nuclear SL-IR 24Sα rDNA [135] GPI [114] kDNA COII [54]	[97]
Amapá, Brazil (1996)	Presumed oral, but no vehicle mentioned, 17 family members/neighbors, no deaths	Parasite hemocultures (8)	TcI (n = 2) TcIII or IV (ZIII) (n = 6)	Nuclear SL-IR [43]	[93]
Amapá, Amazonas and Pará, Brazil	Presumed oral, but unclear if from outbreaks or sporadic	Parasite hemocultures (19)	TcI (n = 14) TcIV (n = 5)	Nuclear ssrDNA [267] RAPD [341] kDNA Cytb [62]	[95]
Six different locations, Colombia (1992–2010)	Attributed to unspecified food/drink Tibú n = 24, 1992; Guamal n = 13, 1999; Lebrija n = 10, two deaths, 2008; Bucaramanga, n = 5, one death, 2009; San Vicente, 2010; Aguachica, n = 12, 2010	50 biological clones from eight isolates (1 Tibú, 2 Lebrija, 3 Bucaramanga, 2 San Vicente de Chucurí)	TcI (n = 49) TcIV (n = 1)	Nuclear SL-IR [48] 24Sα rDNA Microsatellites (24 loci) [70] kDNA mtMLST (10 loci) [317]	[251]
Aguachica, Colombia (2010)	Presumed oral, but no vehicle mentioned, 11 confirmed cases	Parasite hemoculture (1)	TcI (n = 1)	Nuclear SL-IR [50]	[252]
Littoral, French Guiana (2005)	Palm fruit juice, eight infected families, no deaths	Parasite hemocultures (6)	TcI (n = 6)	Nuclear SL-IR 24Sα rDNA [135]	[240]
Caracas, Venezuela (2007)	Guava juice served in a school, 103 infected patients, one death	Parasite hemocultures (3)	TcI (n = 3)	Nuclear SL-IR [41] 24Sα rDNA [50]	[250]
Chichiriviche and Antimano, Venezuela (2009 and 2010)	Common meals (food not identified), suspected/confirmed: 85/33, 35/15, no deaths	Parasite hemocultures (42)	TcI (n = 42)	Nuclear Microsatellites (23 loci) [70]	[245]
Five different locations, Venezuela	Five of the six reported outbreaks in Venezuela, no additional details given	Parasite hemocultures (28)	TcI (n = 28)	**Nuclear** SL-IR 24Sα rDNA 18S rRNA [135]	[254]

^†^Refers to number of clinical samples genotyped to DTU-level only, not total cohort size. DTU: Discrete typing unit; kDNA: Kinetoplast DNA; MLEE: Multilocus enzyme electrophoresis; mtMLST: Maxicircle multilocus sequence typing; RAPD: Random amplification of polymorphic DNA; ND: Not described; SL-IR: Spliced-leader intergenic region.

Most investigations occur months after the outbreaks and consist of vector and animal reservoir studies in the area of the outbreak [243,95,98,245,254], based on the assumption that outbreak vehicles were contaminated by infected triatomine feces or anal gland secretions of infected opossums, which contain infective trypomastigotes [255]. The investigation in the area of the Santa Catarina outbreak (typed in humans as TcII) demonstrated TcI in opossums and both TcI and TcII in triatomine vectors, implicating local triatomines as the most likely source of infection [243].

Recent laboratory data suggest that parasite contact with host gastric acid may render trypomastigotes more invasive through changes in parasite surface glycoproteins, and that this interaction may underlie the increased clinical severity observed in orally acquired Chagas disease [256–258]. The *T. cruzi* surface glycoprotein gp82 is highly resistant to proteolysis and has been shown to mediate migration through the stomach mucin layer and invasion of gastric mucosal cells. Different parasite strains express distinct isoforms of gp90, a second surface glycoprotein, which present differential susceptibility to digestion by pepsin. Strains with pepsin-digested gp90 have highly efficient gastric mucosal invasion and establish patent *T. cruzi* infections, in contrast to those with pepsin-resistant gp90 which invade poorly. Experimental infection with a Colombian TcI isolate demonstrated efficient oral and intraperitoneal transmission, in contrast to a Peruvian strain (TcV) which was much less efficient by the oral than the intraperitoneal route [259]. Similarly, a strain from the 2005 Santa Catarina outbreak (TcII) was highly invasive, producing a robust infection and high mortality in a mouse model, unlike CL Brener (TcVI) and G (TcI) strains, with these differences attributed to the pepsin susceptibility of the gp90 isoforms of the strains [256].

## *T. cruzi* lineage in Chagas disease diagnostics and chemotherapy

Diagnosis of Chagas disease in the chronic phase is based on serological detection of anti-*T. cruzi* IgG antibodies. However, no single assay has sufficient sensitivity and specificity to be used alone; confirmed diagnosis relies on concordant results from at least two tests using different antigens and/or formats (usually ELISA, indirect immunofluorescence and/or indirect hemagglutination) [260]. Differential sensitivities to serodiagnostic tests have been reported between Bolivia and Peru, where two different commercial rapid tests based on recombinant antigens demonstrated sensitivities of 87.5 and 90% versus 30 and 54%, respectively [261]. Rapid test sensitivities were closely correlated with absorbance values on whole parasite lysate-based ELISAs. Similarly, low sensitivities of recombinant antigen ELISA and rapid tests have also been reported from Panama [262,263] and Mexico [264]. No clear correlation between serodiagnostic test reactivity and local *T. cruzi* DTU has been observed; instead, these discrepancies may reflect weaker adaptive immune responses to parasite antigens between endemic populations [265].

Current treatment options for Chagas disease are limited to benznidazole and nifurtimox. While both drugs have high cure rates in the acute phase, efficacy during the chronic phase has been much harder to document, largely due to the lack of a timely, sensitive test of cure [18,266]. Two recent trials have successfully employed quantitative real-time PCR to rapidly detect treatment failure of the new drug candidates posaconazole and E1224, a related drug [268–271]. In contrast, fewer than 10% of those who completed the 60-day benznidazole course had positive results by PCR during the follow-up period. The posaconazole trial required positive pretreatment results by PCR as a prerequisite for enrollment, while the E1224 trial was preceded by an optimization exercise that achieved 92% sensitivity for PCR using multiple specimens and optimized techniques. These two trials provide strong support for the use of PCR as a primary outcome measure in clinical trials in the chronic phase. No data on *T. cruzi* strain are currently available from these trials, but parasite diversity was unlikely to be high because the E1224 trial was conducted in Bolivia, and nearly all patients treated in the posaconazole trial in Spain were also of Bolivian origins.

Small human studies, clinical impression and findings in animal models tentatively suggest that parasite susceptibility varies with geographical location and parasite lineage [272–275] as might be expected given the crucial role played by some genetic loci (e.g., *TcNTR*) in resistance to both benznidazole [276] and nifurtimox [277]. However, direct human data are sparse and *in vitro* epimastigote assays have poor correlation with *in vivo* mouse models of drug response [278]. Natural drug resistance to either drug has been reported in both patient-derived isolates as well as sylvatic unexposed strains [273,279]. The most comprehensive *in vivo* analysis of parasite susceptibility was conducted in the 1980s using 47 parasite strains isolated from patients, sylvatic reservoirs and vectors, and subsequently inoculated into mice [273]. In mouse models, cure rates to both benznidazole and nifurtimox were close to 100% for parasite strains from Argentina and the southernmost region of Brazil, where the predominant DTUs are presumed to be TcV and TcVI. In central and Atlantic Brazil, where TcII is likely to be more common, cure rates ranged from 50 to 65%, while strains from sylvatic reservoir hosts and vectors showed highly variable responses [273].

The inability to dissect the relative contribution of parasite genetics to treatment failure, disease pathology and progression represents a major hurdle to the assessment of novel drug candidates. The advent and optimization of a non-invasive, *in vivo*, bioluminescent imaging system which can facilitate real-time monitoring of chronic parasite burden has the potential to address these key questions using current and prospective chemotherapies, prior to human evaluations [280,281].

## Expert commentary & five-year view

Establishing or excluding any relationship between *T. cruzi* genetic diversity and clinical outcome will require significant improvements in study design and reporting, patient sampling and parasite genotyping (Box 1). As encountered throughout this article, meta-analyses of historical studies are impeded by the paucity (and in many cases, complete absence) of descriptive clinical data among genotyping publications. Use of a standardized CCM stratification method (or supplemental data to allow reclassification) would facilitate comparison between studies. Because of the long asymptomatic period before the onset of clinical signs and symptoms, cross-sectional evaluations will inevitably misclassify some individuals as IND who will later develop CCM; if parasite genetics contribute to the later progression of disease, this misclassification will introduce a bias toward the null hypothesis (‘parasite genetics are not associated with CCM’). This bias will be exacerbated if CCM and IND groups are not matched by age distribution; without deliberate age-matching (individual or group frequency-matching), the IND group will always be younger than the symptomatic group due to the natural history of the disease [25]. Only one genotyping study ensured that the age distributions of IND and CCM groups were comparable [189]; no other publications in this review took this potential confounder into account. Cohort studies of Chagas cardiomyopathy are rare, but could provide extremely valuable opportunities to examine whether *T. cruzi* strain affects the risk of development and progression, while avoiding the biases of cross-sectional studies. The strict clinical criteria used in the two most prominent current cohorts (the control arm of the BENEFIT trial [282] and the REDS-II study [27]) should provide a firm basis for interpretation, although thus far, neither has published *T. cruzi* genotypic data.

The pathogenesis of Chagas cardiomyopathy is widely believed to be multifactorial, with contributions from both parasite and host genetics; evaluating parasite genotype in isolation is therefore likely inadequate. The addition of immunology, human genetics and more detailed epidemiological evaluations could provide a richer assessment of the possible interactions of factors modifying clinical outcome. Similarly, studies of congenital Chagas disease could be made more rigorous by ensuring that both mother and infant specimens are tested, and that the source population, criteria for and age at congenital diagnosis are more clearly detailed in publications. The maternal immune response clearly plays an essential role in modulating congenital transmission risk, and optimally would be evaluated in tandem with parasite strain diversity [224,225]. Finally, the impact of vector control initiatives, including changes in force of infection and circulating strain compositions, needs to be considered, particularly when comparing genotypic data from the same endemic area over time.

Epidemiological as well as genotype data are extremely sparse for GI Chagas disease. The widely accepted observation that gastrointestinal Chagas disease is much rarer in Central and northern South America than in the Southern Cone still rests on clinical reports rather than quantitative population-based epidemiological studies. In part, this stems from the impracticality of performing barium studies in field conditions and on large numbers of people. A study in Bolivia used clinical swallowing time (by placing a stethoscope on the neck, asking the subject to swallow and timing the duration) to assess prevalence of early esophageal changes; 22% of seropositive participants had swallowing times longer than the clinically established cut-off of 10 s, compared to none of the seronegative participants [283]. If field-friendly methods such as swallowing times can be validated by comparison with barium studies, this could provide a useful tool for epidemiological assessments in conjunction with parasite genetic analysis.

Many articles are difficult to interpret because the relationship between data by specimen and by patient is not explicit. Synthesis of the literature as a whole is challenging because multiple manuscripts by the same authors often present genotyping data from samples from the same source population, without specifying the degree of overlap [197,198,284]. Re-characterization of the same specimens in consecutive papers may inflate the prevalence estimates of the resulting DTUs. For all of these limitations, more transparent data presentation would be invaluable. The current ‘open-access’ movement [193] may assist in this area, and could be enhanced by the use of common identifiers between datasets from the same source population to facilitate such meta-analyses.

Modifications to study design must be complemented by parallel improvements in clinical *T. cruzi* genotyping. As previously discussed, parasite isolation by hemoculture, xenodiagnoses or animal inoculation is not ideal and introduces unquantifiable biases; novel methodologies to enrich *T. cruzi* DNA in clinical specimens and circumvent loss of clonal diversity during parasite culturing stages warrant further investigation. Crucially, the current repertoire of clinical genotyping techniques is restricted to DTU-level classification and insufficient to explore the potential interaction between parasite multiplicity of infection and clinical outcome. Illumina amplicon sequencing, recently developed to explore intra-host pathogen genetic diversity, involves the generation of millions of ‘short’ sequencing reads from individual samples, potentially allowing correlation of read depth with genotype abundance [285–288]. This strategy has been used to examine intra-patient multiclonality among chronic Chagas disease patients, across the clinical spectrum (asymptomatic to severe cardiomyopathy, megaesophagus or megacolon) in Goiás, Brazil and matched mother–infant pairs from Cochabamba, Bolivia. While no relationship between parasite multiclonality and patient sex, age or clinical symptoms has been observed thus far, putative evidence of diversifying selection affecting antigenic genes was detected, suggesting a link between genetic diversity in this gene family and survival in the mammalian host [289].

As yet unidentified, diverse genetic characteristics of *T. cruzi* may influence clinical outcome. However our systematic review demonstrates no unequivocal evidence for an association between *T. cruzi* genotype and chronic morbidity, risk of reactivation, or congenital or oral transmission. In recent publications, the most consistent finding is that specimens from all groups reflect the predominant genotypes circulating in the local area. Results from patients with reactivation indicate that mixed infections may be the rule, rather than the exception. Further elucidation of the role of *T. cruzi* genotype in disease pathogenesis will require improvements in both study design and genotyping techniques.

Box 1. **Recommendations to improve assessments of the interaction between *Trypanosoma cruzi* genotype and clinical disease status.**

**Clinical and epidemiological data**

- Use standardized cardiomyopathy stratification scheme and/or provide supplemental data to allow reclassification
- Present data on specific electrocardiogram and echocardiogram abnormalities used to define Chagas cardiomyopathy
- Present data on age; individual or frequency-matching by age to reduce bias
- Test as representative a sample of cohort or other study participants as possible
- Include data collection on other potentially confounding risk factors such as vector exposure history, immunological response and human genetics
- Incorporate reliable DTU identification into ongoing and future cohort studies of cardiomyopathy and congenital *Trypanosoma cruzi* transmission
- Develop and validate methods for identification of gastrointestinal Chagas disease adaptable to population-based studies

**Data transparency**

- Explicit presentation of the relationship between specimens and patients across publications
- Open-access datasets delinked from personal identifiers, but maintaining specimen linkage from one study to another

**Application of laboratory techniques**

- Use of standardized *T. cruzi* nomenclature, genotyping protocols, shared reference strains, and central, open-access repositories of genotypic data
- Genotyping using at least two independent markers to increase sensitivity, facilitate unequivocal DTU resolution and mitigate potential misclassification due to parasite recombination

**Improved laboratory techniques**

- Techniques for unambiguous DTU classification, particularly between TcII, TcV and TcVI
- Development of novel methods to enrich parasite DNA sufficiently to enable genotyping directly from low-parasite load clinical specimens

DTU: Discrete typing unit.

## Notes

Key issues

- *Trypanosoma cruzi* displays remarkable genetic diversity, which is believed to contribute to the biological, epidemiological and clinical variation observed among Chagas disease foci.
- *T. cruzi* strains can be classified into six stable genetic lineages known as discrete typing units (TcI–TcVI), with distributions loosely defined by geography, ecology and transmission cycle.
- Establishing an association between *T. cruzi* genotype and clinical outcome is primarily complicated by low peripheral parasitemia in chronic infections.
- Current evidence suggests that parasite strains detected in patients with or without morbidity reflect the principal lineage circulating among domestic transmission cycles in that geographical area.
- Likewise, local parasite genotypes are frequently implicated in acute *T. cruzi* oral outbreaks, which have been increasingly reported in recent years.
- Loss of immunological control through immunosuppression or HIV co-infection allows sequestered parasites to return to the circulation, thus providing the best indicator of the complexity of natural infections.
- Women who transmit *T. cruzi* to one child are more likely to transmit to other offspring, suggesting that parasite genotype may be one factor influencing the risk of vertical infection. However, to date, the discrete typing units isolated from mothers and infants reflect the principal lineages circulating in that area.
- Improvements in both study design and genotyping techniques are required to advance our understanding of the role of *T. cruzi* genotype in disease pathogenesis.
